# A Methodological and Reporting Quality Assessment of Systematic Reviews/Meta-Analyses about Chinese Medical Treatment for Gastroesophageal Reflux Disease

**DOI:** 10.1155/2020/3868057

**Published:** 2020-09-24

**Authors:** Zipan Lyu, Zhongyu Huang, Fengbin Liu, Zhengkun Hou

**Affiliations:** ^1^Graduate College, Guangzhou University of Chinese Medicine, Guangzhou Guangdong, China; ^2^Integrated Chinese and Western Medicine Postdoctoral Research Station, Jinan University, Guangzhou Guangdong, China; ^3^Gastroenterology Department, The First Affiliated Hospital, of Guangzhou University of Chinese Medicine, Guangzhou Guangdong, China

## Abstract

**Objective:**

To access the methodological and reporting quality of systematic reviews (SRs)/meta-analyses (MAs) about Chinese medical treatment for gastroesophageal reflux disease (GERD).

**Methods:**

The PubMed, Wanfang Data, China National Knowledge Infrastructure (CNKI), Chinese Science and Technology Periodical Database (VIP), Chinese Biomedical (CBM), Web of Science, and Cochrane Library databases were searched from inception to June 2020. Two researchers independently screened the literature considering the eligibility criteria. Overview Quality Assessment Questionnaire (OQAQ), Assessment of Multiple Systematic Reviews 2 (AMSTAR 2), and Preferred Reporting Items for Systematic Reviews and Meta-Analyses (PRISMA) guidelines were used to assess the methodological and reporting quality of the included reports. The Grading of Recommendations Assessment, Development and Evaluation (GRADE) system was used to evaluate the level of evidence in each report.

**Results:**

Thirty-three SRs/MAs met the inclusion criteria. The OQAQ results showed that defects in the methodological quality of 17/32 reports were major, with scores of 3 points. Analyzing a single item as the object, search strategies (item 2), and risk of bias in individual studies (item 4) was considered poor. The AMSTAR 2 results showed that 25.4% of the items were not reported, and 7.8% of the items were only partially reported. The overall assessment of AMSTAR 2 showed the majority of systematic reviews and meta-analyses were of low/very low (31/33, 93.9%) methodological quality, with a lack of protocol registration and excluded study list. The PRISMA results showed that 19.9% of items were not reported, and 15.2% of items were only partially reported, due to a lack of protocol registration and study selection methods. The methodological and reporting quality of the included studies was generally poor. Evidence evaluation with GRADE showed that most (31/33) of the included studies had low or very low levels of evidence.

**Conclusion:**

The methodological and reporting quality of SRs/MAs about Chinese medical treatment for GERD is generally poor. The main problems included incomplete search strategies, risk of bias in individual studies, the lack of protocol registration and excluded study list, and incorrect study selection methods.

## 1. Introduction

Gastroesophageal reflux disease (GERD) is a global chronic disease that affects 10% to 20% of Europeans and 2.5% to 7.1% of Asians [1–3]. However, mainstream therapy approaches including acid suppression therapy, prokinetics, and antireflux surgery were reported to be with little efficacy in relieving reflux symptoms [4]. To improve individual quality of life, patients with GERD seek alternative medicines for reducing the frequency and severity of reflux symptoms such as heartburn and belching [5]. As empirical alternative approaches, it was reported that traditional Chinese medicine (TCM) therapies such as acupuncture and herb treatment are safe and efficient in the treatment of gastroesophageal reflux disease. However, adequate high-quality evidence from normatively designed clinical trials is still in lack of further promotion of these approaches and their basic theory [6].

Summarizing empirical evidence that fits prespecified eligibility criteria with the application of statistical methods and systematic review/meta-analysis (SR/MA) provides multilevel evidence as reference for clinical decision-making. More remarkably, the conclusion from these works could be of varying quality which significantly depends on the material and process of the researches. Therefore, it is important to make a proper evaluation of existing resources of SR/MA for the achievement of reliable evidence among ambiguous even contradictory conclusions. And the promotion of the research about the evaluation of the quality of SR/MA is necessary for further application of TCM approaches as a treatment for complex diseases such as GERD [7, 8].

In the past decades, methodologies and instruments had been designed and developed as assisting tools for the evaluation of reliability and credibility of the SR/MA among which the Overview Quality Assessment Questionnaire (OQAQ), A Measurement Tool to Assess Systematic Reviews (AMSTAR), The Preferred Reporting Items for Systematic Reviews and Meta-Analyses (PRISMA) guidelines, and The Grading of Recommendations Assessment, Development and Evaluation (GRADE) were reported to be widely used for evaluation of the quality of researches and conclusion they are drawn.

The OQAQ (containing 10 items) was developed by Oxman and Guyatt in 1991 to evaluate the quality of SR/MA methodologies [9]. The AMSTAR containing 11 items was jointly developed by clinical epidemiologists at the Medical Research Centre of the University of Vrije Universiteit, the Netherlands, and the University of Ottawa, Canada, in 2007. The tool was updated as the AMSTAR 2 (including 16 items) in 2017 for evaluating SR/MA methodological quality [10, 11]. The PRISMA guideline (containing 27 items) was developed based on the Quality of Reporting of Meta-Analyses (QUOROM) statement, which was developed by an international team represented by David Moher, and this was revised in 2009 to guide and evaluate the writing of systematic reviews [12, 13]. The GRADE is an evidence rating system created by the GRADE Working Group. It was officially launched in 2004 and is used to evaluate the quality of evidence and the level of recommendation [14]. To provide reliable evidence for TCM treatment about GERD in clinical practice, this study made use of the OQAQ, AMSTAR 2 scale, and PRISMA guidelines for accessing the methodological and reporting quality of the SRs/MAs about Chinese medical treatment for GERD with the hope of further reminding clinicians to be more cautious about the quality of SRs/MAs on GERD.

## 2. Materials and Methods

### 2.1. Search Strategy

Two trained researchers independently searched eight databases, including the PubMed, Wanfang Data, China National Knowledge Infrastructure (CNKI), Chinese Science and Technology Periodical Database (VIP), Chinese Biomedical (CBM), Web of Science, and Cochrane Library databases were searched from inception to June 2020. The following keywords and combined free words were used in the search: “gastric esophageal reflux disease,” “Chinese medical therapy,” “systematic review,” and “meta-analysis.” The grammar was adjusted according to different databases. The reference list of each included paper was screened to find potentially relevant articles that were not initially identified by the search. If there were multiple updates for a study, only the latest version would be included.

### 2.2. Inclusion Criteria

One study would be included if the following conditions were met: (1) the study was self-labeled or designed as a systematic review, meta-analysis, or systematic review and meta-analysis; (2) the studies included participants who were diagnosed with GERD (reflux oesophagitis (RE) or nonerosive reflux disease (NERD) or Barrett's esophagus (BE)); (3) the topic was set as the treatment of gastroesophageal reflux disease with Chinese medicine; (4) these articles were full texts that have been published.

### 2.3. Exclusion Criteria

Publications were excluded according to the following criteria: (1) studies were not SRs/MAs; (2) studies did not concern a clinical question regarding human beings; (3) studies had missing or unclear data for final analysis.

### 2.4. Data Extraction

Two trained researchers independently extracted data from the included SRs/MAs with preestablished forms. To ensure the validity of the data extraction form, 10% of the literature samples were randomly pretested and appropriate modifications would be made to the forms based on the preassessment results. The following basic characteristics of the included SRs/MAs were extracted: (1) the first author, (2) the study country, (3) the year of publication, (4) the language of publication, (5) the numbers of authors, (6) the type of study, (7) the numbers of participants, (8) the numbers of included RCTs, (9) the invention, (10) comparisons, (11) bias risk assessment tool results, (12) funding sources, and (13) Cochrane review.

### 2.5. Assessment of Methodological and Reporting Quality

The OQAQ and the AMSTAR 2 scale were used to evaluate the methodological quality, and the PRISMA guidelines were used to access the quality of reporting for the included SRs/MAs. According to the matching level between the content of the report and item of those three scales, the score of each item was 0, 0.5, or 1, and the weight of each field was equal. When it was clear that the conditions were met, the item would be judged as “complete reporting (score 1),” and when it was clear that the conditions were not met, the item would be judged as “no reporting (score 0).” When the item was related but not fully described, the item would be judged as “partial reporting (score 0.5).” The OQAQ questionnaire results were set as follows when evaluating the methodological quality of a single SR/MA: 1 = obvious deficiencies, 3 = major defects, 5 = minor defects, and 7 = negligible defects. For each item of the three scales, the reporting rate was calculated as the sum of the score of each article and presented as a percentage to analyze problems with the SR/MA methodologies and reporting quality. Besides, the included SRs/MAs would be grouped into three preset subgroups, including language, funding, and Cochrane review, to explore potential factors that may affect the quality of SR/MA methodology and reporting. Each SR/MA was independently evaluated by two authors, and differences were resolved through discussion.

### 2.6. Assessment of Quality of the Evidence

GRADE was used to assess the SR/MA level of evidence. The quality of the evidence was classified into four levels: high, moderate, low, and very low. The GRADE system assessment results were combined with the methodological and reporting quality assessment results to produce GERD drug treatment recommendations.

## 3. Results

### 3.1. Search Results

**Figure 1:**
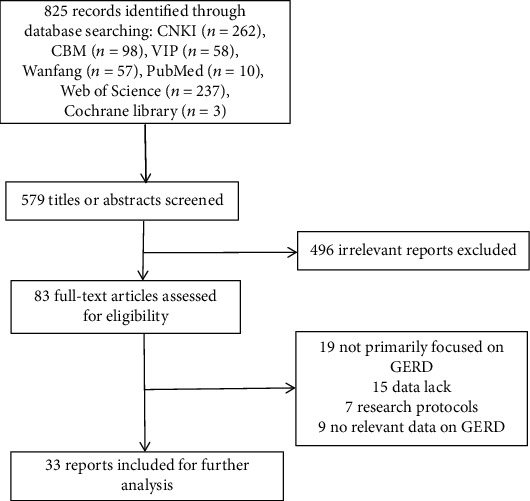
PRISMA flow diagram for the selection process for SRs/MAs about Chinese medical treatment for GERD.

The search protocol identified 825 studies with potential relevance. After screening titles and abstracts, 72 studies were eligible for full-text review, and 39 studies were excluded according to the exclusion criteria. Finally, 33 reports [15–46], including 22 network meta-analyses (NMA)/MAs, 6 SRs, and 5 NMAs/MAs and SRs, underwent quality assessment. Information on the search protocol is shown in Figure 1.

### 3.2. General Characteristics

There were 28 reports [15–24, 26–32, 34, 35, 38–45] in Chinese and 5 reports [25, 33, 36, 37, 46] in English. 18 studies reported the sources of funding; but none of them were Cochrane reviews. The 33 studies included 124 RCTs and 15686 GERD patients and compared the efficacies of multiple Chinese medical interventions. All 33 studies have used the risk of bias assessment tool; only 1 study [23] used a tool but did not describe it, and the other 32 studies used the Jadad scale, Cochrane handbook, or both of them as the risk of the bias assessment tool. The details of all the included reports are shown in Table 1.

### 3.3. Methodological Quality

The results of the OQAQ and AMSTAR 2 scale for evaluating methodological quality are shown in Tables 2 and 3. The evaluation results of the OQAQ showed that the defects in the methodological quality of 16 reports were negligible, with scores of 7 points, and 17 reports had major defects, with scores of 3 points. Analyzing a single item as the object, search strategies (item 2), and risk of bias in individual studies (item 4) was considered poor according to the OQAQ, with a no reporting rate of 36.8% and a partial reporting rate of 25%. The remaining items (1, 3, 5-9), such as information sources (item1), study selection (item3), and synthesis of results (item 5), in the OQAQ, had a complete reporting rate of 100%. According to the AMSTAR 2 results, the patient, intervention, comparison, outcome (PICO) criteria (item 1), explanation of study design (item 3), included studies (item 8), combined data (item 11), and satisfactory explanation (item 14) had been completely reported with the rate of 100%; protocol registration (item 2), the listing of excluded studies (item 7), and funding (item 10) had been incompletely reported with rates of 0%, 31.3%, and 31.3%, respectively. A single study was used as the object for analysis; the AMSTAR 2 scale showed that on average, each study had a complete reporting rate of 66.8%, a partial reporting rate of 7.8%, and a no reporting rate of 25.4%.

### 3.4. Reporting Quality

The results of the PRISMA quality evaluation are shown in Table 4. The average score for the 33 studies was 19.5 out of 27 points, suggesting that the quality of the included studies was low. A single study was used as the object for analysis; the highest score was 25 points [46], the lowest score was 15 points [45], and there was a large gap in quality between studies. The no reporting rate for PRISMA results was 19.9%, and the partial reporting rate was 15.2%. In the single-item analysis, the reporting rates for the topic (item 1), the rationale (item 3), and the synthesis of results (item 21) were 100% (33/33). Structured summary (item 2), eligibility criteria (item 6), information sources (item7), summary measures (item 13), study selection (item 17), and synthesis of results (item 21) were completely or partially reported. None of the studies reported that protocol registration (item 5), study selection (item 9), data items (item 11), and additional (item 16) were poorly reported.

### 3.5. Evidence Quality

The results of the evidence quality assessment are shown in Table 5. There was no high-quality level of study. Most of the included studies had either a low or very low-quality level, with only two studies [35, 46] having a moderate level of evidence.

### 3.6. Subgroup Analysis

Since there were no Cochrane reviews, subgroup analyses were conducted only for topics about language and funding. The results of the two subgroup analyses according to language and funding are shown in Table 6. Methodological quality assessments with the OQAQ and AMSTAR 2 scale suggested that the results of the funding subgroups were similar. According to the PRISMA results, 28 reports [15–24, 26–32, 34, 35, 38–45] in Chinese had an average quality score of 19.2, and 5 reports [25, 33, 36, 37, 46] in English had an average quality score of 24. The 16 studies [17, 20, 22, 24, 26–29, 31, 33, 35, 37, 40–45] that received funding had an average quality score of 19.6, and the nonfunded research had an average quality score of 20.2. The two studies with moderate evidence levels were nonfunded studies according to the evaluation results of GRADE.

## 4. Discussion

This study showed that the progress of SR/MA research regarding Chinese medical treatment of GERD was not good. The methodological and reporting quality of the related studies was low. The results of the OQAQ showed that the defects in the methodological quality of 16 reports were negligible, with scores of 7 points, and 17 reports had major defects, with scores of 3 points. The results of the AMSTAR 2 scale showed that the unreported rate was 25.4% and the partial reporting rate was 7.8%, totaling 66.8%. The no reporting rate for PRISMA results was 19.9%, and the partial reporting rate was 15.2%. The above defects reduced the credibility of relevant conclusions to some extent.

### 4.1. Characteristics of Research Scope and Results

Since the inclusion of SRs/MAs in this study did not consider the use of the Cochrane criteria, it is not comparable to the quality differences reported by those who applied Cochrane and non-Cochrane methodologies. However, some MAs [47–49] reported excellent methodological quality according to the Cochrane criteria, indicating that rigorous expert-led methods training and expert collaboration guidance are conducive to the production of high-quality MAs.

The results reported by all of the studies were positive which may be related to the fact that positive results are more often published than negative results. By applying systemic analysis, Young et al. [50] found that “if a study reports a negative result, then publication is quite difficult”. Zhu et al. [51] reported in an article that “negative findings of MAs can be independent factors for improved methodologies (a 0.6-point increase in the AMSTAR score).” Therefore, it is important when a study produces a negative result, it should be reported honestly and not disregarded or modified.

### 4.2. Subgroup Analysis

Funded research was inferior to nonfunded research in both reporting and methodological quality, and it was also found that funded research tended to be published in Chinese (14/16). It is a common phenomenon in China that well-known research institutions or researchers are more likely to receive funding in relevant fields. Likewise, journals tend to favor research from well-known institutions or individuals to improve their reputation instead of evaluating research based on quality. This is in line with Reingewertz and Lutmar [52] who noted that academic in-group bias is general. This discovery reminds researchers about the necessity of making an objective evaluation of each study. When performing an SR/MA, it is important to search as comprehensively as possible, be cautious about language and country restrictions, and include good research to improve the overall quality of the study.

### 4.3. Methodological Quality

With the OQAQ applied for evaluating the quality of the included research methods, the main reasons leading to the decrease in scores were the literature search strategies (item 2) and the risk of individual study bias (item 4). SR/MA is a secondary analysis, and a large number of studies have shown that incomplete search strategies will generate selection bias and affect the quality of studies. Though the search strategy about researches was considered on the three scales, there are differences among their requirements. For the OQAQ, it is required that researchers should include both electronic searches and manual searches while only electronic searches were required in the PRISMA guidelines and AMSTAR 2 scale. However, with the development of interworking technology and the application of databases, there have been more approaches for researchers to obtain potentially relevant research, such as the preprint websites http://arxiv.org/ and https://www.biorxiv.org/, which allow researchers to obtain grey literature which is a kind of important and valuable information recourse. The China Journal Full-text Database (CJFD) includes 6,100 core journals and important journals in various disciplines. Since 1994, the data integrity of the 6,100 periodicals has reached 98%. Therefore, it should be realized that the use of an electronic search alone would not affect the credibility of the final results. In this study [40], the methodological quality assessment score was reduced from 7 to 3 points due to the lack of manual searching. Manual searching is very important, but whether it is necessary or not is debated at present since the OQAQ was developed in 1991 and it seems to be slightly out of date. Moreover, it does not involve the evaluation of publication bias and conflicts of interest even though publication bias is an extremely important indicator of the methodological quality of systemic evaluation. By performing a systematic review, Pussegoda et al. [53] showed that the OQAQ has been used significantly less by researchers than the PRISMA guidelines or the AMSTAR scale. This article would recommend the application of the AMSTAR scale for the evaluation of the methodological quality of SRs/MAs. For researchers who want to produce SRs/MAs, a comprehensive search strategy with a combination of search databases, professional websites, professional internal conference proceedings, libraries, clinical trial registration platforms, and official registration websites of relevant institutions would be recommended.

According to the design of AMSTAR 2, items 2, 4, 7, 9, 11, 13, and 15 were critical for methodological quality evaluation. However, none of the studies provided protocol registration information (item 2) or a listing of excluded studies (item 7). The reason why the selected articles failed to provide preliminarily excluded studies maid is that the excluded list is merely required in the current guidance for research reporting and publishing out of the scope of research. Especially for domestic periodicals in China, due to the limitations of the layout and word counts, editors cannot list relatively lengthy exclusion documents in the manuscript. Therefore, the author generally believes that the magazine editors or peer reviewers will ask the author to exclude the list of documents during the manuscript review stage and conduct a detailed assessment before the paper is published. As to the fifth item in PRISMA guidelines, it is required for the report about protocol registration of the research. Lesley et al. [54] regarded systematic reviews as a form of observational research in which a protocol registered before the start of the study would help to ensure its scientific validity and feasibility. Registration can effectively control bias that may occur in all aspects of research and prevent researchers from arbitrarily changing the research protocol. Booth et al. [55] developed the perspective that a registered plan can effectively reduce the duplication of SRs/MAs and reduce scientific research resource waste. In 2011, Tricco et al. [56], an international expert in evidence-based medicine, jointly called for the registration of all meta-analyses and systematic reviews worldwide. This would enable highly scientific, rigorous, and transparent production processes and increase the publication rate of scientific articles. As to registered websites, the Cochrane Collaboration and PROSPERO would be recommended for research registration. The difference between the two registration systems is that the PROSPERO platform accepts studies with a broad scope with a relatively simple and easy process for registration and auditing while that of the Cochrane Collaboration is more complicated and restrict.

### 4.4. Reporting Quality

As shown in the result of the PRISMA evaluation, more than 80% of items were not completely reported. As to study selection of the articles, there were 87.6% (29/33) of enrolled articles that did not completely report it in the method section while only 24.2% (8/33) of enrolled articles did not completely report it in the result section. For reporting the study selection method, item 9 requires that the report includes the selection process for the study, and item 17 requires that the results of each step in the screening process be reported. There were obvious differences in the reporting rates for items 9 and 17, in which some scholars believe are related to expensive publication fees. However, by comparing the report lengths of the highest (J. Li et al. [45]) and lowest scoring (S. Li et al. [46]), the number of words was reduced by rationally using typesetting, cleverly combining tables, and adding less-important content to the appendix, while still ensuring the rigor and integrity of the report.

### 4.5. GRADE Evidence Assessment

In the GRADE assessment, most of the evidences included in the study were low-level or very low-level evidence. The main reason for the degradation in the level of evidence was that included studies had a high risk of bias. This indicates that the quality of RCTs related to drug treatment of GERD is poor, making it difficult to provide support for evidence-based medicine. Multicenter, large-sample RCTs are urgently needed. Researchers should focus on high-quality, relevant clinical research in the future. There were two studies (JY.K. Dai et al. [33], S. Li et al. [46]) with intermediate levels of evidence; their PRISMA scores were 24.5 and 25, respectively. Supported by the strength of the same evidence, Li et al.'s study [46] had the best reporting quality and was closest to fulfilling the requirements of the Cochrane Collaboration. High-quality SRs/MAs have level I evidence for evidence-based medicine. It is well known that the Cochrane Collaboration's SR/MA criteria have been widely used in guidelines because of their rigorous and scientific development. They are known to the academic community as the gold standard for evidence-based results. Because of the absence of relevant Cochrane reviews and because two studies had the same level of evidence according to GRADE, we creatively introduced PRISMA evaluation results and recommended the conclusions of Li et al. [46] to clinical decision-makers. However, given the possible biases, a further systematic review is needed.

### 4.6. Limitations

There are several limitations to the design of the research. First of all, since self-labeled other than the standardized definition of system review was set as the inclusion criterion for literature selection, there may be a predetermined low score in the quality of the report. Secondly, due to the unquantified item setting of the AMSTAR scale and PRISMA scale and different understandings from evaluators, subjective bias was introduced resulting in differences in the final scoring results. Thirdly, the scope of analysis as limited to the SRs/MAs of TCM in treating GERD. Those studies around the therapies such as PPI and other mainstream treatments were not included; therefore, the extrapolation of the conclusions was limited.

## 5. Conclusion

The methodological and reporting quality of SRs/MAs about Chinese medical treatment for GERD is generally poor. The main problems included incomplete search strategies, risk of bias in individual studies, the lack of protocol registration and excluded studies list, and incorrect study selection methods.

## Figures and Tables

**Table 1:** Characteristics of the included studies.

First author	Country	Year	Language	Number of authors	Study type	Participants (*n*)	Number of included studies	Intervention	Comparison	Risk of the bias assessment tool	Funding	Cochrane review
Song [15]	China	2008	Chinese	4	SR	772	10	Xiao Xian Xiong decoction	Modern medicine	Jadad	No	No
Yang [16]	China	2011	Chinese	1	SR	2074	25	Herbal formula of TCM	Modern medicine	Cochrane	No	No
Zhao [17]	China	2011	Chinese	5	SR	772	21	Herbal formula with liver soothing and stomach therapy	Modern medicine	Jadad	Yes	No
Chen [18]	China	2014	Chinese	2	MA	1138	13	Ban Xia Hou Po decoction alone or in combination with modern medicine	Modern medicine	Cochrane	No	No
Sun [19]	China	2014	Chinese	3	SR	1703	14	Herbal formula with Tong Jiang He Wei therapy	Modern medicine	Jadad	No	No
Zhang [20]	China	2014	Chinese	1	SR	587	11	Herbal formula of TCM	Modern medicine	Cochrane	Yes	No
Wang [21]	China	2014	Chinese	1	SR	1895	29	Herbal formula of TCM	Modern medicine	Cochrane	No	No
Li [22]	China	2015	Chinese	3	SR & MA	1068	11	Herbal formula with Xin Kai Ku Jiang therapy	Modern medicine	Cochrane	Yes	No
Pei [23]	China	2015	Chinese	1	MA	896	10	Herbal formula of TCM	Modern medicine	No description	No	No
Guo [24]	China	2015	Chinese	2	MA	914	12	Ban Xia Xie Xin decoction	Modern medicine	Cochrane	Yes	No
Ling [25]	China	2015	English	10	SR & MA	2706	33	Wen Dan decoction	Conventional therapies such as proton-pump inhibitors, GI motility-promotion drugs, and gastric mucosal protective drugs	Cochrane	No	No
Zheng [26]	China	2016	Chinese	2	MA	2603	34	Chai Hu Shu Gan powder	Modern medicine alone/combined with Chinese medicine	Cochrane	Yes	No
Zheng [27]	China	2016	Chinese	2	MA	1305	11	Ban Xia Hou Po decoction alone or in combination with modern medicine	Modern medicine	Cochrane	Yes	No
Ge [28]	China	2016	Chinese	4	MA	756	6	Herbal formula of TCM	Modern medicine	Jadad	Yes	No
Zhu [29]	China	2017	Chinese	9	MA	3706	30	External treatment of TCM	Modern medicine	Cochrane	Yes	No
Ghung [30]	China	2017	Chinese	1	MA	894	11	Acupuncture	Modern medicine	Cochrane	No	No
Chen [31]	China	2017	Chinese	3	MA	2002	26	Ban Xia Xie Xin decoction	Modern medicine	Cochrane and Jadad	Yes	No
Chen [32]	China	2017	Chinese	3	MA	684	9	Herbal formula of TCM	Modern medicine alone/combined with Chinese medicine	Cochrane	No	No
Dai [33]	China	2017	English	6	SR & MA	1068	12	Modified Ban Xia Xie Xin decoction	Modern medicine	Cochrane	Yes	No
Li [34]	China	2018	Chinese	5	SR & MA	1158	18	Acupuncture and herbal formula	Modern medicine	Cochrane	No	No
Xie [35]	China	2018	Chinese	4	MA	1047	14	External treatment of TCM	Modern medicine	Jadad	Yes	No
Xiao [36]	China	2018	English	8	MA	1444	14	Herbal formula of TCM	Modern medicine	Cochrane and Jadad	No	No
Zhu [37]	China	2018	English	1	SR & MA	1235	12	Acupuncture	Modern medicine	Cochrane	Yes	No
Li [38]	China	2019	Chinese	6	NMA	1262	16	External treatment of TCM	Modern medicine	Jadad	No	No
Xie [39]	China	2019	Chinese	6	NMA	1667	21	Herbal formula of TCM	Modern medicine	Jadad	No	No
Fu [40]	China	2019	Chinese	9	MA	864	10	Herbal formula with liver soothing and stomach therapy	Modern medicine	Jadad	Yes	No
Li [41]	China	2019	Chinese	9	MA	1163	11	Acupuncture	Modern medicine	Cochrane	Yes	No
Song [42]	China	2019	Chinese	4	MA	1135	18	Acupuncture or and herbal formula of TCM	Modern medicine	Cochrane	Yes	No
Wu [43]	China	2019	Chinese	6	MA	2155	26	Acupuncture	Modern medicine	Cochrane	Yes	No
Xiao [44]	China	2019	Chinese	6	MA	3132	26	Xuan Fu Hua Dai Zhe Shi decoction	Modern medicine	Jadad	No	No
Song [44]	China	2020	Chinese	4	NMA	2266	26	Herbal formula of TCM	Modern medicine	Cochrane	Yes	No
Li [45]	China	2020	Chinese	3	MA	524	7	Herbal formula with Bu Qi Sheng Yang therapy	Modern medicine	Jadad	Yes	No
Li [46]	China	2020	English	10	MA	966	13	Si Ni Zuo Jin decoction	Modern medicine	Cochrane	No	No

Notes: TCM (traditional Chinese medicine) included herbal formula, acupuncture, and moxibustion; modern medicine included PPIs (proton pump inhibitors), H2RAs (H2 receptor antagonists), and antibiotics.

**Table 2:** The results of OQAQ assessments.

Study	Information sources	Search	Study selection	Risk of bias in individual studies	Synthesis of results	Study selection	Synthesis of results	Limitations	Conclusions	Scores
Song [15]	1	0	1	1	1	1	1	1	1	3
Yang [16]	1	1	1	1	1	1	1	1	1	7
Zhao [17]	1	0	1	0.5	1	1	1	1	1	3
Chen [18]	1	0	1	1	1	1	1	1	1	3
Sun [19]	1	0	1	1	1	1	1	1	1	3
Zhang [20]	1	1	1	0.5	1	1	1	1	1	7
Wang [21]	1	1	1	1	1	1	1	1	1	7
Li [22]	1	0	1	0.5	1	1	1	1	1	3
Pei [23]	1	0	1	0.5	1	1	1	1	1	3
Guo [24]	1	1	1	0.5	1	1	1	1	1	7
Ling [25]	1	1	1	1	1	1	1	1	1	7
Zheng [26]	1	0	1	1	1	1	1	1	1	3
Zheng [27]	1	0	1	1	1	1	1	1	1	3
Ge [28]	1	1	1	0.5	1	1	1	1	1	7
Zhu [29]	1	1	1	0.5	1	1	1	1	1	7
Ghung [30]	1	1	1	0.5	1	1	1	1	1	7
Chen [31]	1	0	1	0.5	1	1	1	1	1	3
Chen [32]	1	0	1	0.5	1	1	1	1	1	3
Dai [33]	1	1	1	1	1	1	1	1	1	7
Li [34]	1	0	1	0.5	1	1	1	1	1	3
Xie [35]	1	0	1	0.5	1	1	1	1	1	3
Xiao [36]	1	1	1	1	1	1	1	1	1	7
Zhu [37]	1	1	1	0.5	1	1	1	1	1	7
Li [38]	1	0	1	0.5	1	1	1	1	1	3
Xie [39]	1	1	1	0.5	1	1	1	1	1	7
Fu [40]	1	0	1	1	1	1	1	1	1	3
Li [41]	1	0	1	0.5	1	1	1	1	1	3
Song [42]	1	1	1	0.5	1	1	1	1	1	7
Wu [43]	1	1	1	0.5	1	1	1	1	1	7
Xiao [44]	1	0	1	0.5	1	1	1	1	1	3
Song [44]	1	1	1	0.5	1	1	1	1	1	7
Li [45]	1	0	1	0.5	1	1	1	1	1	3
Li [46]	1	1	1	1	1	1	1	1	1	7

Notes: 0 = no reporting; 0.5 = partial reporting; 1 = complete reporting; score (1 = obvious deficiencies; 3 = major defects; 5 = minor defects; 7 = negligible defects).

**Table 3:** The results of AMSTAR 2 assessments.

Study	Q1	Q2	Q3	Q4	Q5	Q6	Q7	Q8	Q9	Q10	Q11	Q12	Q13	Q14	Q15	Q16	Rank	Yes [*n* (%)]	Partial yes [*n* (%)]	No [*n* (%)]
Song [15]	1	0	1	0.5	0	1	0	1	0	1	1	0	0	1	0	1	Very low	8 (50%)	1 (6.3%)	7 (43.8%)
Yang [16]	1	0	1	0.5	1	1	0	1	1	0	1	1	1	1	1	0	Very low	11 (68.8%)	1 (6.3%)	4 (25%)
Zhao [17]	1	0	1	0.5	0	1	0.5	1	1	0	1	1	1	1	0	0	Very low	9 (56.3%)	2 (12.5%)	5 (31.3%)
Chen [18]	1	0	1	0.5	0	1	0	1	1	0	1	1	1	1	1	0	Very low	10 (62.5%)	1 (6.3%)	5 (31.3%)
Sun [19]	1	0	1	0.5	0	0	0	1	0	0	1	0	0	1	1	1	Very low	8 (50%)	1 (6.3%)	7 (43.8%)
Zhang [20]	1	0	1	0.5	0	1	0	1	1	0	1	1	1	1	1	1	Very low	11 (68.8%)	1 (6.3%)	4 (25%)
Wang [21]	1	0	1	0.5	1	1	0.5	1	1	0	1	1	1	1	1	0	Low	11 (68.8%)	2 (12.5%)	3 (18.8%)
Li [22]	1	0	1	0.5	0	0	0	1	0	0	1	0	0	1	1	1	Very low	8 (50%)	1 (6.3%)	7 (43.8%)
Pei [23]	1	0	1	0	0	0	0	1	1	0	1	1	1	1	1	1	Very low	10 (62.5%)	0 (0%)	6 (37.5%)
Guo [24]	1	0	1	0.5	1	1	0	1	1	0	1	1	1	1	1	0	Very low	11 (68.8%)	1 (6.3%)	4 (25%)
Ling [25]	1	0	1	0.5	0	0	0.5	1	1	1	1	1	1	1	1	1	Moderate	12 (75%)	2 (12.5%)	2 (12.5%)
Zheng [26]	1	0	1	0.5	1	1	0	1	1	1	1	1	1	1	1	0	Low	12 (75%)	1 (6.3%)	3 (18.8%)
Zheng [27]	1	0	1	0.5	0	1	0.5	1	1	0	1	1	1	1	1	1	Low	11 (68.8%)	2 (12.5%)	3 (18.8%)
Ge [28]	1	0	1	0.5	0	1	0	1	1	0	1	1	1	1	1	1	Low	11 (68.8%)	1 (6.3%)	4 (25%)
Zhu [29]	1	0	1	0.5	0	1	0	1	1	0	1	1	1	1	1	0	Low	10 (62.5%)	1 (6.3%)	5 (31.3%)
Ghung [30]	1	0	1	0.5	0	0	0.5	1	1	1	1	1	1	1	1	1	Low	12 (75%)	2 (12.5%)	2 (12.5%)
Chen [31]	1	0	1	0.5	0	1	0.5	1	1	0	1	1	1	1	1	1	Low	11 (68.8%)	2 (12.5%)	3 (18.8%)
Chen [32]	1	0	1	0	0	0	0	1	1	0	1	1	1	1	1	1	Very low	10 (62.5%)	0 (0%)	6 (37.5%)
Dai [33]	1	0	1	0.5	1	1	0.5	1	1	1	1	1	1	1	1	1	Low	13 (81.3%)	2 (12.5%)	1 (6.3%)
Li [34]	1	0	1	0.5	0	1	0	1	1	0	1	1	1	1	1	0	Low	10 (62.5%)	1 (6.3%)	5 (31.3%)
Xie [35]	1	0	1	0.5	0	1	0.5	1	1	0	1	1	1	1	0	0	Low	9 (56.3%)	2 (12.5%)	5 (31.3%)
Xiao [36]	1	0	1	0.5	1	1	0	1	1	1	1	1	1	1	1	1	Low	13 (81.3%)	1 (6.3%)	2 (12.5%)
Zhu [37]	1	0	1	0.5	1	1	0.5	1	1	1	1	1	1	1	1	1	Low	13 (81.3%)	2 (12.5%)	1 (6.3%)
Li [38]	1	0	1	0.5	1	1	0.5	1	1	1	1	1	1	1	1	1	Low	13 (81.3%)	2 (12.5%)	1 (6.3%)
Xie [39]	1	0	1	0.5	1	1	0.5	1	1	1	1	1	1	1	1	1	Low	13 (81.3%)	2 (12.5%)	1 (6.3%)
Fu [40]	1	0	1	0.5	0	0	0	1	0	0	1	0	0	1	1	1	Very low	8 (50%)	1 (6.3%)	7 (43.8%)
Li [41]	1	0	1	0	0	0	0	1	1	0	1	1	1	1	1	1	Very low	10 (62.5%)	0 (0%)	6 (37.5%)
Song [42]	1	0	1	0.5	1	1	0	1	1	0	1	1	1	1	1	0	Very low	11 (68.8%)	1 (6.3%)	4 (25%)
Wu [43]	1	0	1	0.5	0	1	0.5	1	1	0	1	1	1	1	1	1	Low	11 (68.8%)	2 (12.5%)	3 (18.8%)
Xiao [44]	1	0	1	0.5	0	1	0	1	1	0	1	1	1	1	1	0	Low	10 (62.5%)	1 (6.3%)	5 (31.3%)
Song [44]	1	0	1	0.5	1	1	0.5	1	1	0	1	1	1	1	1	0	Low	11 (68.8%)	2 (12.5%)	3 (18.8%)
Li [45]	1	0	1	0.5	0	1	0.5	1	1	0	1	1	1	1	0	0	Low	9 (56.3%)	2 (12.5%)	5 (31.3%)
Li [46]	1	0	1	0.5	1	1	0.5	1	1	1	1	1	1	1	1	1	Moderate	13 (81.3%)	2 (12.5%)	1 (6.3%)
Yes [*n* (%)]	33 (100%)	0 (0%)	33 (100%)	0 (0%)	12 (36.4%)	25 (75.8%)	0 (0%)	33 (100%)	29 (87.9%)	10 (30.3%)	33 (100%)	29 (87.9%)	29 (87.9%)	33 (100%)	29 (87.9%)	20 (60.6%)				
Partial yes [*n* (%)]	—	0 (0%)	—	30 (90.9%)	—	—	15 (45.5%)	0 (0%)	0 (0%)	—	—	—	—	—	—	—				
No [*n* (%)]	0 (0%)	33 (100%)	0 (0%)	3 (9.1%)	21 (63.6%)	8 (24.2%)	18 (54.5%)	0 (0%)	4 (12.1%)	23 (69.7%)	0 (0%)	4 (12.1%)	4 (12.1%)	0 (0%)	4 (12.1%)	13 (39.4%)				
∗ [*n* (%)]	—	—	—	—	—	—	—	—	0 (0%)	—	—	—	—	—	—	—				
@ [*n* (%)]	—	—	—	—	—	—	—	—	0 (0%)	—	—	—	—	—	—	—				
# [*n* (%)]	—	—	—	—	—	—	—	—	—	—	0 (0%)	—	—	—	—	—				
¥ [*n* (%)]	—	—	—	—	—	—	—	—	—	—	—	0 (0%)	—	—	0 (0%)	—				

Notes: Q1 = PICO criteria; Q2 = protocol; Q3 = explanation of study design; Q4 = search; Q5 = study selection in duplicate; Q6 = data extraction in duplicate; Q7 = excluded studies; Q8 = included studies; Q9 = risk of bias; Q10 = funding; Q11 = combined data; Q12 = the potential impact of RoB; Q13 = accounting for RoB; Q14 = satisfactory explanation; Q15 = publication bias; Q16 = conflict of interest; Q2, 4, 7, 9, 11, 13, and 15 are the critical domains; 1 = complete reporting; 0.5 = partial reporting; 0 = no reporting, ∗ = only NRSIs; @ = only randomized controlled trials; # = no meta − analysis; ¥ = no meta − analysis included.

**Table 4:** The result of PRISMA assessments.

Study	Title	Structured summary	Rationale	Objectives	Protocol and registration	Eligibility criteria	Information sources	Search	Study selection	Data collection process	Data items	Risk of bias in individual studies	Summary measures	Synthesis of results	Risk of bias across studies	Additional analyses	Study selection	Study characteristics	Risk of bias within studies	Results of individual studies	Synthesis of results	Risk of bias across studies	Additional analysis	Summary of evidence	Limitations	Conclusions	Funding	Scores
Song [15]	1	0.5	1	0.5	0	0.5	0.5	1	0	1	0.5	1	1	1	1	0	1	1	0	1	1	1	1	0.5	1	1	0	19
Yang [16]	1	0.5	1	0.5	0	1	0.5	1	0	1	1	1	1	1	1	0	1	1	1	1	1	1	1	1	1	1	1	22.5
Zhao [17]	1	0.5	1	0.5	0.5	0.5	0.5	0.5	0	0	0	1	1	0.5	1	1	0.5	0.5	0.5	1	1	1	1	0	1	1	0	17
Chen [18]	1	0.5	1	0.5	0	0.5	1	0.5	1	0.5	1	0.5	1	1	0	0	1	0	1	1	1	0	1	1	0	1	0	17
Sun [19]	1	0.5	1	0.5	0	0.5	0.5	1	0	1	0	1	1	1	1	0.5	1	1	0	1	1	1	1	0	1	1	0	18.5
Zhang [20]	1	0.5	1	1	1	1	1	1	0	1	1	0	1	1	1	0	1	1	0.5	1	1	1	0	1	1	1	1	22
Wang [21]	1	0.5	1	0.5	0	1	1	1	0	1	1	1	1	1	1	1	1	1	1	1	1	1	1	1	1	1	0	23
Li [22]	1	0.5	1	0.5	0	0.5	0.5	1	0	1	0	1	1	1	1	0	1	1	0	1	1	1	0.5	0.5	1	1	0	18
Pei [23]	1	0.5	1	0.5	0	1	1	0.5	0.5	0	0	1	1	1	1	0	1	0	0.5	0	1	1	1	1	0.5	1	1	18
Guo [24]	1	0.5	1	0.5	0	1	1	0.5	0.5	0.5	0	1	1	1	1	1	0.5	1	1	1	1	1	1	1	1	1	0	21
Ling [25]	1	0.5	1	0.5	0.5	1	0.5	1	0	1	1	1	1	1	1	0	1	1	1	1	1	1	1	1	1	1	1	23
Zheng [26]	1	0.5	1	0.5	0	1	1	1	0	1	0	1	1	1	1	0	1	1	1	1	1	1	0	1	1	1	0	20
Zheng [27]	1	0.5	1	0.5	0	0.5	0.5	0	0	0	0	1	1	0.5	1	1	0.5	1	1	1	1	1	1	0	1	1	0	17
Ge [28]	1	1	1	1	0.5	0.5	0	1	1	0.5	1	0	1	1	1	0	1	1	0	1	1	1	0	1	1	1	1	20.5
Zhu [29]	1	0.5	1	1	0.5	0.5	0.5	1	1	1	1	0.5	1	1	1	0	1	1	0	1	1	1	0	1	1	1	1	21.5
Ghung [30]	1	0.5	0.5	1	0	1	0.5	1	0.5	1	1	0.5	1	1	1	0	1	1	1	1	1	1	1	1	1	1	1	22.5
Chen [31]	1	0.5	1	0.5	0.5	1	1	0.5	0.5	0.5	1	1	1	1	0	1	1	1	0.5	1	1	1	1	1	1	0.5	0	21
Chen [32]	1	0.5	1	0.5	0	0	0.5	1	0	1	0	1	1	1	1	0	1	1	0	1	1	1	1	0.5	1	1	0	18
Dai [33]	1	0.5	1	0.5	1	1	1	1	0.5	1	1	1	1	1	1	1	1	1	1	1	1	1	1	1	1	1	0	24.5
Li [34]	1	0.5	1	0.5	0	0	1	1	0	1	1	0.5	1	1	0.5	0	0.5	1	0.5	1	1	0	0.5	1	1	1	1	18.5
Xie [35]	1	0.5	1	0.5	0	1	1	1	0	0.5	1	1	1	1	0	0	0.5	1	1	0.5	1	0	1	0.5	0.5	0	1	17.5
Xiao [36]	1	0.5	1	0.5	0.5	1	0.5	1	0.5	1	1	1	1	1	1	0	1	1	1	1	1	1	1	1	1	1	1	23.5
Zhu [37]	1	0.5	1	0.5	1	1	1	1	0.5	1	1	1	1	1	1	1	0.5	1	1	1	1	0.5	0.5	1	1	1	1	24
Li [38]	1	0.5	1	0.5	0	1	1	1	0	0.5	1	0	1	0	0.5	0	1	0	1	1	1	1	0	0	0.5	0.5	1	16
Xie [39]	1	0.5	1	0.5	0	1	1	0.5	0	0.5	1	1	1	1	0.5	1	1	1	1	0	1	0	1	1	1	1	1	20.5
Fu [40]	1	0.5	1	1	0	1	1	1	0	1	1	0.5	1	1	1	0	0.5	1	0	1	1	1	0	1	1	1	1	20.5
Li [41]	1	0.5	1	0.5	0	1	1	1	0	0.5	1	0	1	0	0	0	1	0	1	1	1	1	0	0	0.5	0.5	1	15.5
Song [42]	1	0.5	1	0.5	0	0.5	0.5	1	0	1	0	1	1	1	1	0	1	1	0	1	1	1	1	0.5	1	1	0	18.5
Wu [43]	1	0.5	1	0.5	0	0.5	1	1	0	1	1	0	1	1	1	0	0.5	1	0	1	1	1	0	1	1	1	1	19
Xiao [44]	1	0.5	1	0.5	0	1	1	0.5	0.5	0.5	0	1	1	1	1	0	1	0	0.5	0	1	1	1	1	0.5	1	1	18.5
Song [44]	1	0.5	1	0.5	0	1	1	0.5	0.5	0.5	0	1	1	1	1	1	1	1	0	1	1	1	1	1	1	0.5	1	21
Li [45]	1	0.5	1	0.5	0	1	1	1	0.5	0	1	0	1	0	0	0	1	0	0.5	1	1	1	0	0	0.5	0.5	1	15
Li [46]	1	0.5	1	0.5	0	1	1	1	1	1	1	1	1	1	1	1	1	1	1	1	1	1	1	1	1	1	1	25
Yes *n* (%)	33 (100%)	0 (0%)	33 (100%)	5 (15.2%)	0 (0%)	22 (66.6%)	20 (60.6)	24 (72.8%)	4 (13.3%)	19 (57.6%)	22 (66.7%)	22 (66.7%)	33 (100%)	28 (8.8%)	25 (75.7%)	23 (69.7%)	25 (75.7%)	26 (78.8%)	16 (48.7%)	29 (87.9%)	33 (100%)	29 (87.9%)	21 (63.6%)	22 (66.6%)	26 (78.8%)	27 (81.8%)	20 (60.6%)	
Partial yes *n* (%)	0 (0%)	33 (100%)	0 (0%)	14 (84.8%)	0 (0%)	9 (27.3%)	12 (36.4%)	8 (24.2%)	10 (30.1%)	10 (30.1%)	0 (0%)	5 (15.2%)	0 (0%)	2 (6.1%)	3 (9.1%)	1 (3.0%)	8 (24.2%)	1 (3.0%)	7 (21.2%)	1 (3.0%)	0 (0%)	1 (3.0%)	3 (9.1%)	5 (15.2%)	6 (18.2%)	5 (15.2%)	0 (0%)	
No *n* (%)	0 (0%)	0 (0%)	0 (0%)	0 (0%)	33 (100%)	2 (6.1%)	1 (3.0%)	1 (3.0%)	19 (57.6%)	4 (13.3%)	11 (33.3%)	6 (18.2%)	0 (0%)	3 (9.1%)	5 (15.2%)	9 (27.3%)	0 (0%)	6 (18.2%)	10 (30.1%)	3 (9.1%)	0 (0%)	3 (9.1%)	9 (27.3%)	6 (18.2%)	1 (3.0%)	1 (3.0%)	13 (39.4%)	

Notes: 1 = complete reporting; 0.5 = partial reporting; 0 = no reporting.

**Table 5:** GRADE evidence profile.

No. of studies	Study design	Risk of bias	Inconsistency	Indirectness	Imprecision	Other considerations	Impact	Certainty	Importance
First author: J.S. Song									
10	Randomized trials	Serious	Serious	Serious	Serious	Publication bias strongly suspected	Most information was obtained from studies with a high risk of bias.	⨁◯◯◯ Very low	Not important
First author: L. Yang									
25	Randomized trials	Serious	Not serious	Not serious	Serious	Publication bias strongly suspected Strong associations of all plausible residual confounding could reduce the demonstrated effect	Most information was obtained from studies with a low or unclear risk of bias, without direct evidence of the outcome.	⨁⨁◯◯ Low	Not important
First author: S.W. Chen									
13	Randomized trials	Serious	Serious	Not serious	Serious	Publication bias strongly suspected	Most information was obtained from studies with a high risk of bias.	⨁◯◯◯ Very low	Not important
First author: C.H. Sun									
14	Randomized trials	Serious	Very serious	Serious	Serious	Publication bias strongly suspected	Most information was obtained from studies with a high risk of bias.	⨁◯◯◯ Very low	Not important
First author: M. Zhang									
11	Randomized trials	Serious	Not serious	Not serious	Serious	Publication bias strongly suspected Strong associations of all plausible residual confounding could reduce the demonstrated effect	Most information was obtained from studies with a low or unclear risk of bias, without direct evidence of the outcome.	⨁⨁◯◯ Low	Not important
First author: Y.Q. Wang									
29	Randomized trials	Serious	Not serious	Not serious	Serious	Publication bias strongly suspected Strong associations of all plausible residual confounding could reduce the demonstrated effect	Most information was obtained from studies with a low or unclear risk of bias, without direct evidence of the outcome.	⨁⨁◯◯ Low	Not important
First author: Y.J. Li									
11	Randomized trials	Serious	Serious	Serious	Serious	Publication bias strongly suspected	Most information was obtained from studies with a high risk of bias.	⨁◯◯◯ Very low	Not important
First author: Y.Q. Fei									
10	Randomized trials	Serious	Serious	Serious	Serious	Publication bias strongly suspected	Most information was obtained from studies with a high risk of bias.	⨁◯◯◯ Very low	Not important
First author: Z.L. Guo									
12	Randomized trials	Serious	Serious	Not serious	Serious	Publication bias strongly suspected	Most information was obtained from studies with a high risk of bias.	⨁◯◯◯ Very low	Not important
First author: W. Ling									
33	Randomized trials	Not serious	Not serious	Not serious	Not serious	Publication bias strongly suspected Strong associations of all plausible residual confounding could reduce the demonstrated effect	Heterogeneity analysis of one subgroup showed that there was obvious statistical heterogeneity among the studies (*I*_2_ = 42.4%, *P* = 0.096).	⨁⨁⨁◯ Moderate	Important
First author: M. Zheng									
34	Randomized trials	Serious	Not serious	Not serious	Serious	Publication bias strongly suspected Strong associations of all plausible residual confounding could reduce the demonstrated effect	Most information was obtained from studies with a low or unclear risk of bias, without direct evidence of the outcome.	⨁⨁◯◯ Low	Not important
First author: M. Zheng									
11	Randomized trials	Serious	Not serious	Not serious	Serious	Publication bias strongly suspected Strong associations of all plausible residual confounding could reduce the demonstrated effect	Most information was obtained from studies with a low or unclear risk of bias, without direct evidence of the outcome.	⨁⨁◯◯ Low	Not important
First author: Y.H. Guo									
6	Randomized trials	Serious	Serious	Serious	Serious	Publication bias strongly suspected	Most information was obtained from studies with a high risk of bias.	⨁◯◯◯ Very low	Not important
First author: J.J. Zhu									
30	Randomized trials	Serious	Not serious	Not serious	Serious	Publication bias strongly suspected Strong associations of all plausible residual confounding could reduce the demonstrated effect	Most information was obtained from studies with a low or unclear risk of bias, without direct evidence of the outcome.	⨁⨁◯◯ Low	Not important
First author: H.K. Ghung									
11	Randomized trials	Serious	Not serious	Not serious	Serious	Publication bias strongly suspected Strong associations of all plausible residual confounding could reduce the demonstrated effect	The true effect may be substantially different from the estimated effect.	⨁⨁◯◯ Low	Not important
First author: J.K. Chen									
26	Randomized trials	Serious	Serious	Serious	Serious	Publication bias strongly suspected	Most information was obtained from studies with a high risk of bias.	⨁◯◯◯ Very low	Not important
First author: K.H. Chen									
9	Randomized trials	Serious	Serious	Serious	Serious	Publication bias strongly suspected	Most information was obtained from studies with a high risk of bias.	⨁◯◯◯ Very low	Not important
First author: Y.K. Dai									
12	Randomized trials	Serious	Not serious	Not serious	Serious	Publication bias strongly suspected Strong associations of all plausible residual confounding could reduce the demonstrated effect	Most information was obtained from studies with a low or unclear risk of bias, without direct evidence of the outcome.	⨁⨁◯◯ Low	Not important
First author: G.Y. Li									
18	Randomized trials	Serious	Serious	Serious	Serious	Publication bias strongly suspected	Most information was obtained from studies with a high risk of bias.	⨁◯◯◯ Very low	Not important
First author: J.R. Xie									
14	Randomized trials	Serious	Serious	Serious	Serious	Publication bias strongly suspected	Most information was obtained from studies with a high risk of bias.	⨁◯◯◯ Very low	Not important
First author: J. Xiao									
14	Randomized trials	Serious	Not serious	Not serious	Serious	Publication bias strongly suspected Strong associations of all plausible residual confounding could reduce the demonstrated effect	Most information was obtained from studies with a low or unclear risk of bias, without direct evidence of the outcome.	⨁⨁◯◯ Low	Not important
First author: J.J Zhu									
12	Randomized trials	Serious	Not serious	Not serious	Serious	Publication bias strongly suspected Strong associations of all plausible residual confounding could reduce the demonstrated effect	Most information was obtained from studies with a low or unclear risk of bias, without direct evidence of the outcome.	⨁⨁◯◯ Low	Not important
First author: L.Q. Li									
16	Randomized trials	Serious	Serious	Serious	Serious	Publication bias strongly suspected	Most information was obtained from studies with a high risk of bias.	⨁◯◯◯ Very low	Not important
First author: S. Xie									
21	Randomized trials	Serious	Not serious	Not serious	Serious	Publication bias strongly suspected Strong associations of all plausible residual confounding could reduce the demonstrated effect	The true effect may be substantially different from the estimated effect.	⨁⨁◯◯ Low	Not important
First author: X. Fu									
10	Randomized trials	Serious	Not serious	Not serious	Serious	Publication bias strongly suspected Strong associations of all plausible residual confounding could reduce the demonstrated effect	Most information was obtained from studies with a low or unclear risk of bias, without direct evidence of the outcome.	⨁⨁◯◯ Low	Not important
First author: J.F. Li									
11	Randomized trials	Serious	Serious	Serious	Serious	Publication bias strongly suspected	Most information was obtained from studies with a high risk of bias.	⨁◯◯◯ Very low	Not important
First author: Q.Z. Song									
18	Randomized trials	Serious	Not serious	Not serious	Serious	Publication bias strongly suspected Strong associations of all plausible residual confounding could reduce the demonstrated effect	Most information was obtained from studies with a low or unclear risk of bias, without direct evidence of the outcome.	⨁⨁◯◯ Low	Not important
First author: X.H. Wu									
26	Randomized trials	Serious	Serious	Serious	Serious	Publication bias strongly suspected	Most information was obtained from studies with a high risk of bias.	⨁◯◯◯ Very low	Not important
First author: K.M. Xiao									
26	Randomized trials	Serious	Serious	Serious	Serious	Publication bias strongly suspected	Most information was obtained from studies with a high risk of bias.	⨁◯◯◯ Very low	Not important
First author: Q.Z. Song									
26	Randomized trials	Serious	Not serious	Not serious	Serious	Publication bias strongly suspected Strong associations of all plausible residual confounding could reduce the demonstrated effect	The true effect may be substantially different from the estimated effect.	⨁⨁◯◯ Low	Not important
First author: J. Li									
7	Randomized trials	Serious	Serious	Serious	Serious	Publication bias strongly suspected	Most information was obtained from studies with a high risk of bias.	⨁◯◯◯ Very low	Not important
First author: S.W. Li									
13	Randomized trials	Not serious	Not serious	Not serious	Not serious	Publication bias strongly suspected Strong associations of all plausible residual confounding could reduce the demonstrated effect	Lack of blinding biased the estimates of the treatment effect.	⨁⨁⨁◯ Moderate	Important

**Table 6:** The results of subgroup analysis.

		OQAQ				AMSTAR2			PRISMA	GRADE	
		Score = 1 (*n* (%))	Score = 3 (*n* (%))	Score = 5 (*n* (%))	Score = 7 (*n* (%))	Yes (*n* (%))	Partial yes (*n* (%))	No (*n* (%))	Score	Moderate	Low/very low
Language	Chinese	0 (0%)	17 (40.7%)	0 (0%)	11 (39.3%)	10.1 (63.4%)	1.3 (8.1%)	4.6 (28.5%)	19.2	0 (0%)	28 (100%)
	English	0 (0%)	0 (0%)	0 (0%)	5 (100%)	12.8 (80%)	1.8 (11.3%)	1.4 (8.7%)	24	2 (40%)	3 (60%)

Funding	Funding	0 (0%)	9 (50%)	0 (0%)	9 (50%)	10.4 (64.9%)	1.4 (9%)	4.2 (26.1%)	19.6	0 (0%)	18 (100%)
	Non-funding	0 (0%)	8 (53.3%)	0 (0%)	7 (46.7%)	10.7 (67%)	1.3 (8%)	4 (25%)	20.2	2 (13.3%)	13 (86.7%)

## Data Availability

All related data would be available on request to the corresponding author (fenghou5128@126.com).
